# Characteristics of a Novel Zearalenone Lactone Hydrolase ZHRnZ and Its Thermostability Modification

**DOI:** 10.3390/ijms25179665

**Published:** 2024-09-06

**Authors:** Xinlan Liu, Yanan Wang, Xin Fang, Yu Tang, Gaigai Wang, Yongpeng Guo, Jianmin Yuan, Lihong Zhao

**Affiliations:** 1 State Key Laboratory of Animal Nutrition and Feeding, Poultry Nutrition and Feed Technology Innovation Team, College of Animal Science and Technology, China Agricultural University, No. 2. West Road Yuanmingyuan, Beijing 100193, China; lxl1215629182@163.com (X.L.); wyn@cau.edu.cn (Y.W.); fx18289372269@163.com (X.F.); tangyu@cau.edu.cn (Y.T.); wanggaigai@cau.edu.cn (G.W.); yuanjm@cau.edu.cn (J.Y.); 2 Eyasclub, College of Animal Science and Technology, China Agricultural University, No. 2. West Road Yuanmingyuan, Beijing 100193, China; 3 College of Animal Science and Technology, Henan Agricultural University, Zhengzhou 450046, China; guoyp@henau.edu.cn

**Keywords:** zearalenone, lactone hydrolase, thermostability, mutation

## Abstract

Zearalenone (ZEN) is a toxic secondary metabolite produced by the *Fusarium* fungi, which widely contaminates grains, food, and feed, causing health hazards for humans and animals. Therefore, it is essential to find effective ZEN detoxification methods. Enzymatic degradation of ZEN is believed to be an eco-friendly detoxification strategy, specifically thermostable ZEN degradation enzymes are needed in the food and feed industry. In this study, a novel ZEN lactone hydrolase ZHRnZ from *Rosellinia necatrix* was discovered using bioinformatic and molecular docking technology. The recombinant ZHRnZ showed the best activity at pH 9.0 and 45 °C with more than 90% degradation for ZEN, α-zearalenol (α-ZOL), β-zearalenol (β-ZOL) and α-zearalanol (α-ZAL) after incubation for 15 min. We obtained 10 mutants with improved thermostability by single point mutation technology. Among them, mutants E122Q and E122R showed the best performance, which retained more than 30% of their initial activity at 50 °C for 2 min, and approximately 10% of their initial activity at 60 °C for 1 min. The enzymatic kinetic study showed that the catalytic efficiency of E122R was 1.3 times higher than that of the wild-type (WT). Comprehensive consideration suggests that mutant E122R is a promising hydrolase to detoxify ZEN in food and feed.

## 1. Introduction

Zearalenone (ZEN), also known as F-2 toxin, is an estrogenic mycotoxin produced from the *Fusarium* species, which is widely found in moldy maize, wheat, barley, and other crops [1]. ZEN and its derivatives, including α-ZOL, β-ZOL, α-ZAL, and β-zearalanol (β-ZAL), competitively bind to estrogen receptors, causing reproductive toxicity [2], immunotoxicity [3], cytotoxicity [4], and genotoxicity [5] in animals and humans. Furthermore, previous studies have shown that the estrogenic activity of α-ZOL is three times greater than that of ZEN, while the estrogenic activity of β-ZOL is similar to ZEN [6].

**Figure 1:**
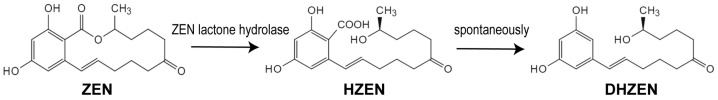
Enzymatic degradation of ZEN by lactone hydrolase.

It is pressing to search for an effective detoxification method to reduce or remove ZEN in matrices. Nowadays, many technologies have been developed to decontaminate ZEN in feed and food, including physical, chemical, and biological methods [7]. It is well known that the microbial and enzymatic detoxification of ZEN has potential application prospect in food and feed due to its high specificity and efficiency, minimal loss of nutrients in the food crop, and lack of secondary pollution [8,9]. According to the reported studies, ZEN-degrading strains can be isolated from the soil, fermented foods, and the gastrointestinal digesta of animals [10]. *Aspergillus niger* FS10 can remove 89.56% of ZEN from potato dextrose broth (PDB) medium [11]. The reported ZEN-degrading enzymes mainly include two classes, oxidase and hydrolase. Bacterial CotA laccase [12] and dye decolorizing peroxidase (BsDyP) [13] can oxidize ZEN to its oxidation products. Kakeya et al. [14] identified the first ZEN lactone hydrolase (ZHD101) from *Clonostachys rosea* IFO 7063. The ZEN lactonase converted ZEN to hydrolyzed ZEN (HZEN) via hydrolyzing the ester bond of its lactone ring, and then the HZEN was spontaneously converted to decarboxylated HZEN (DHZEN) (Figure 1) [15]. The estrogenic activity of HZEN and DHZEN is markedly diminished compared to ZEN [2].

Researchers have discovered many new lactone hydrolases since ZHD101 was reported. Gao et al. [16] identified the activity of the lactonase ZHD_LD from *Exophiala spinifera*, which shares 60.15% of its amino acid identity and a conserved catalytic triad with ZHD101. Hu et al. [17] reported a new lactonase, ZenH from *Aeromicrobium* sp. HA, which has seven times greater activity than ZENG [18]. However, most of the reported lactonases have shown an alkalescent optimal pH and have relatively low thermostability (Table 1), which is unsuitable for further application in the food and feed industry [19]. Therefore, several studies have focused on how to improve the thermostability of lactonases by molecular modification technology. Qiu et al. [20] developed a double-site mutant, V153H-V158F, which showed increased specific activity and thermostability compared to the wild-type ZHD101. A double-site mutant, H134L/S136L, which has an approximately 40% higher residual activity than the wild-type ZENG at 53 °C for 5 min, was constructed by Zhang et al. [18].

In this study, a new potential ZEN lactonase ZHRnZ, which shares a 54.61% amino acid identity with ZHD101, from *Rosellinia necatrix*, was discovered and characterized. Furthermore, the thermostability of ZHRnZ was improved by single-point mutation technology.

## 2. Results and Discussion

### 2.1. Bioinformatics Analysis of ZHRnZ and Its Molecular Docking with ZEN

**Figure 2:**
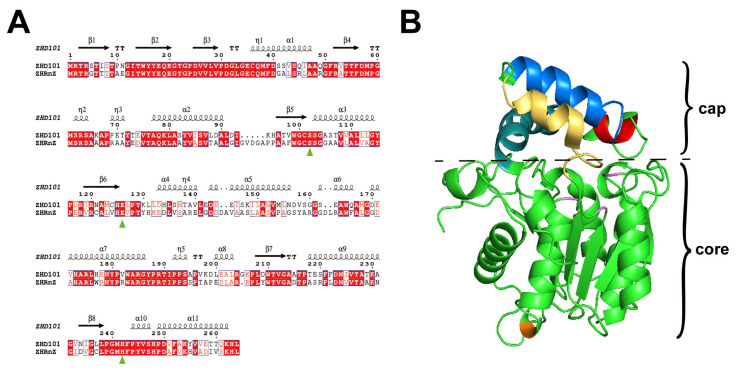
(**A**) Protein sequence alignment between ZHRnZ and ZHD101. The green triangles represent the catalytic triad of ZHRnZ. (**B**) The simulative crystal structure of a subunit of ZHRnZ. Blue, yellow, red, and cyan represent the α-helices 4–7, respectively. Pink represents the catalytic triad. Orange represents the mutation site.

Here, the amino acid sequence of ZHD101 (Accession No. BAC02717.1) was compared by the protein basic local alignment search tool (BLAST) in the National Center for Biotechnology Information (NCBI) database, among which a putative ZEN lactone hydrolase, ZHRnZ (Accession No. GAP90066.2), with a 54.61% homology of ZHD101, from *Rosellinia necatrix*, is a potentially relevant ZEN hydrolase. ZHRnZ, with a theoretical molecular mass and isoelectric point of 28.8 kDa and 5.96, respectively, encodes 272 amino acids. No signal peptide sequence was predicted. The conserved region and catalytic triad of ZHRnZ were predicted by a multiple sequence alignment analysis (Figure 2A). The results showed that there is a conserved catalytic triad, Ser107-His250-Glu131, in ZHRnZ.

The crystal structure of ZHRnZ was simulated through SWISS-MOLEL based on the structure of ZHD101 (PDB ID: 3WZL) as a template. It was proven that ZHRnZ was a homologous dimer with typical cap and core domains in its subunits, and the substrate-binding site existed between the two domains (Figure 2B).

**Figure 3:**
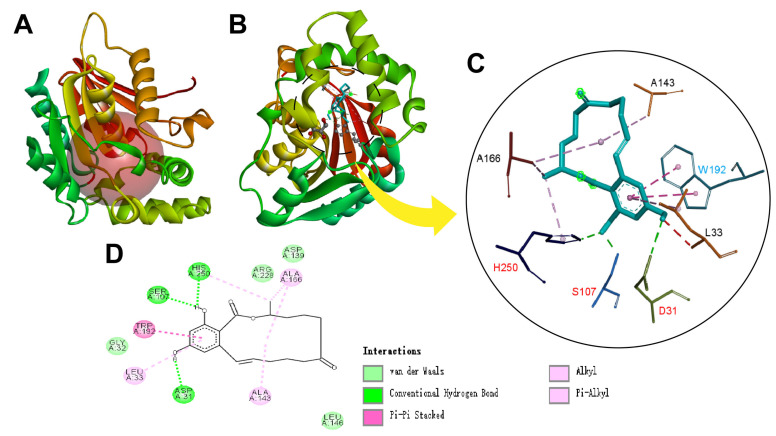
Results of molecular docking between ZHRnZ and ZEN. (**A**) The molecular docking box. Docking site: x = −16.747, y = 17.206, z = 6.303. radius: 11 Å. (**B**) The interaction between ZEN and ZHRnZ. Inside the dotted circle is the catalytic pocket of ZHRnZ. (**C**) Substrate–enzyme interaction networks of ZHRnZ. The red labels represent the amino acid residues involved in forming hydrogen bonds. The blue label represents the amino acid residues involved in forming π–π bonds. The black labels represent other amino acid residues involved in forming hydrophobic forces. (**D**) 2D display of interaction force of substrate-enzyme.

The molecular docking between ZHRnZ and ZEN was performed using AutoDock Vina, with the Ser107 in the catalytic triad as the docking center. The result was visualized using Discovery Studio 2019 (Figure 3A). As shown in Figure 3B, the substrate ZEN was bound to a deep pocket between the core and cap domains, which were adjacent to the catalytic triad Ser107-His250-Glu131 [30]. The two hydroxyl hydrogens in the benzene ring of ZEN formed hydrogen bonds with Aps31, Ser107, and His250 as seen in Figure 3C, D. Gly32, Asp139, Leu146, and Arg228 formed hydrophobic interactions with ZEN, providing a hydrophobic environment for substrate–enzyme binding. Conserved residue Trp192 interacted with the aromatic group of ZEN through π–π bond. Similarly, Trp185 also had a π-π hydrophobic interaction with the aromatic groups of ZEN in ZHD607, which was explained by how the indole plane of Trp185 was perpendicular to the dihydroxybenzoic acid dihydroxyl of ZEN [22]. Zhou et al. [31] suggested that the *p*-π interaction between Trp183 and substrate is responsible for the reactant recognition and allocation in the hydrolysis of ZEN by ZHD. The Trp192 in ZHRnZ should have the same function.

### 2.2. Gene Cloning, Expression and Purification of ZHRnZ

**Figure 4:**
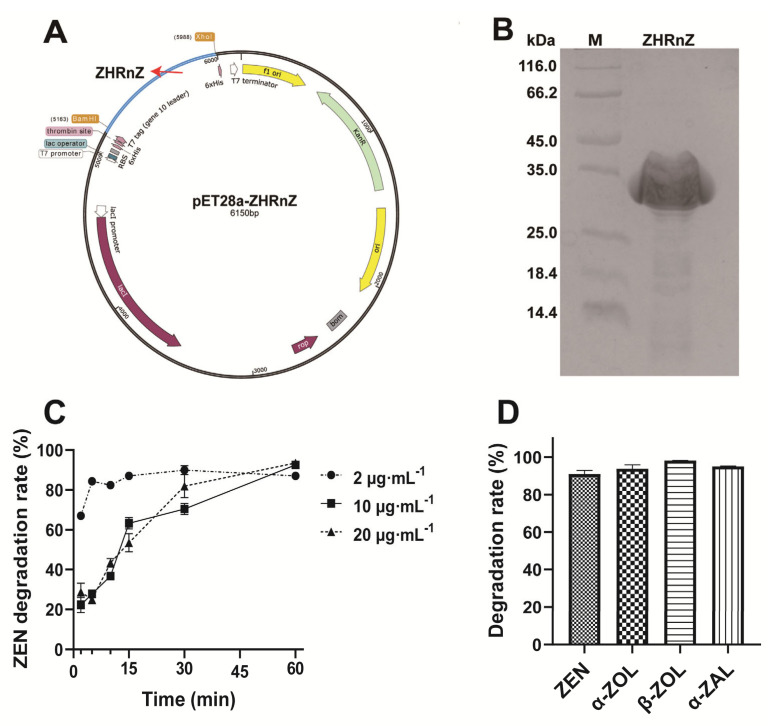
(**A**) The map of recombinant plasmid pET28a-ZHRnZ. The blue part represents the optimized ZHRnZ gene sequence. (**B**) SDS-PAGE analysis of the recombinant ZHRnZ. Lane M represents the protein marker (116.0, 66.2, 45.0, 35.0, 25.0, 18.4 and 14.4 kDa). (**C**) The degradation rate of different concentrations of ZEN using recombinant ZHRnZ. (**D**) The degradation rate of ZEN and its derivatives using recombinant ZHRnZ.

The optimized ZHRnZ gene was inserted in plasmid pET28a between BamHI and Xhol to yield the recombinant plasmid pET28a-ZHRnZ (Figure 4A). The sodium dodecyl sulfate polyacrylamide gel electrophoresis (SDS-PAGE) analysis of the purified recombinant ZHRnZ showed a clear single band with an approximate molecular mass of 28 kDa (Figure 4B).

The degradation rate of different concentrations of ZEN by ZHRnZ (25 μg·mL^−1^) was initially determined at 37 °C, pH 7.5. As shown in Figure 4C, the degradation rate of ZEN (2 μg·mL^−1^, 10 μg·mL^−1^, 20 μg·mL^−1^) was over 80% after being incubated with ZHRnZ for 1 h. Meanwhile, ZHRnZ could degrade more than 90% of ZEN and its three derivatives α-ZOL (10 μg·mL^−1^), β-ZOL (10 μg·mL^−1^), and α-ZAL (10 μg·mL^−1^) at 45 °C, pH 9.0 after incubation for 15 min (Figure 4D). According to the previous studies, the affinity of ZEN and its derivatives to estrogen receptors is in the following order: α-ZOL > α-ZAL > β-ZOL > ZEN > β-ZAL [32]. The toxicity of α-ZOL and α-ZAL was higher than ZEN, and β-ZAL has a similar toxicity to ZEN. Therefore, it is concluded that ZHRnZ has great prospects in detoxifying ZEN derivatives.

### 2.3. Enzymatic Properties of Recombinant ZHRnZ

**Figure 5:**
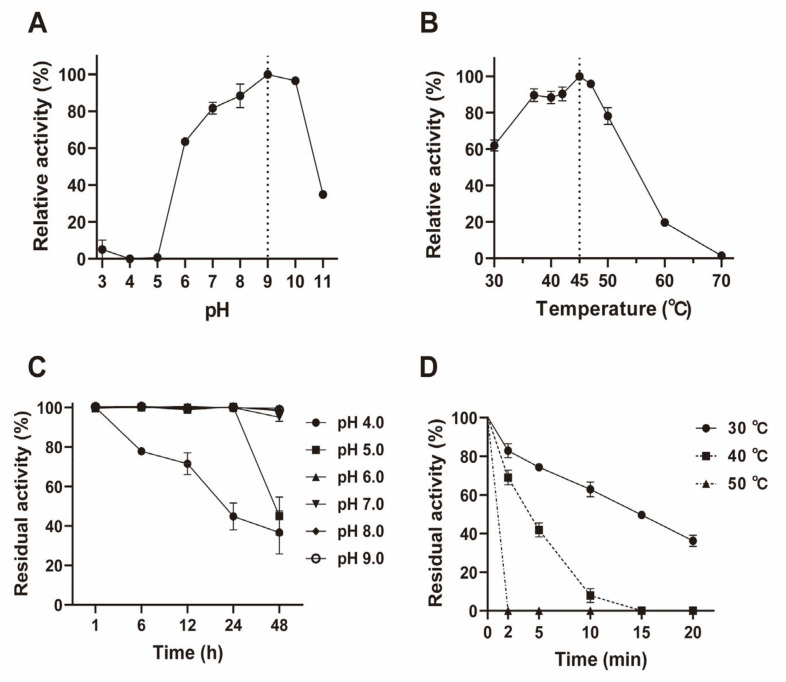
Enzymatic properties of recombinant ZHRnZ. (**A**) The optimal pH of recombinant ZHRnZ for the degradation of ZEN. (**B**) The optimal temperature of recombinant ZHRnZ for the degradation of ZEN. (**C**) The pH resistance of recombinant ZHRnZ for the degradation of ZEN. (**D**) The thermostability of recombinant ZHRnZ for the degradation of ZEN.

It is well known that a weakly acidic pH is vital for the ZEN lactonase when it is applied as a food or feed enzyme. Results showed that the recombinant ZHRnZ had over 60% relative activity during a broad pH range (6.0–10.0), while the optimal pH was 9.0 (Figure 5A). However, the ZHRnZ was completely inactivated when the pH was below 6.0, indicating that acidic conditions could deactivate the enzyme. According to previous studies, ZEN lactonases are mostly neutral and alkalescent hydrolases, and the optimal pH for this kind of enzyme is usually 8.0–9.0 [33]. For example, the optimal pH of lactonases TRI [23] and ZENC [24] were 9.5 and 8.0, respectively. Fortunately, the recombinant ZHRnZ maintained strong stability at pH 6.0–9.0 for 48 h, retaining over 95% of its initial activity as shown in Figure 5C. The recombinant ZHRnZ had over 99% residual activity at pH 5.0 for 24 h but only 43% residual activity for 48 h. At pH 4.0, the recombinant ZHRnZ gradually lost its activity with time increase and showed 44% residual activity for 24 h. It could be seen that ZHRnZ was a considerably stable enzyme in a weakly alkaline or even a weakly acidic environment. Moreover, a study reported that ZEN hydrolase can be mutated to lower the optimal pH for the enzyme to work in the animal’s stomach [34].

The optimal temperature of most reported ZEN lactonases is relatively low, for example, CbZHD (35 °C) [27], ZENG (38 °C) [18], and Zhd11D (35 °C) [28]. The optimal temperature for enzyme activity of recombinant ZHRnZ towards ZEN was observed at 45 °C, and ZHRnZ retained over 60% relative activity at 30–50 °C as shown in Figure 5B. It shows that ZHRnZ has a higher temperature optimum. Thermostability is also an important index to assess whether an enzyme is suitable for the application in food and feed industry [28]. Ideally, the enzyme has strong thermostability and can withstand high temperatures during the granulation process of food and feed. Nevertheless, the reported ZEN lactonases showed poor thermostability generally. For instance, the residual activity of Zhd518 was only 25% at 45 °C for 10 min [29], and ZHD607 retained 56% of its initial activity at 40 °C for 10 min [22]. Similarly, the thermostability of recombinant ZHRnZ was poor and exhibited only 42% residual activity at 40 °C for 5 min, and entirely lost its activity after incubation for 2 min at 50 °C (Figure 5D).

### 2.4. Single Point Mutation Improves the Thermostability of ZHRnZ

As previously mentioned, recombinant ZHRnZ was similar to the most reported ZEN lactonases with poor thermostability, such as CbZHD [27], ZENC [24], Zhd518 [29], ZHD607 [22], ZENG [18], ZHD-P [25], and ZenA [35]. These wild-type enzymes were unsuitable for direct application in heat-treated food and feed. Therefore, many researchers made attempts to improve the thermostability of these kinds of enzymes through molecular modification. Shi et al. [26] performed two N-terminal mutations at ZENY, and the residual activity of the mutants NΔ11 and N5V were increased by approximately 13% and 25%, respectively, at 45 °C for 10 min. Molecular dynamics (MD) simulation can calculate the root mean square fluctuation (RMSF) value which reflects the flexibility of amino acid residues. The higher the RMSF value, the greater the flexibility of the residues. The method of replacing highly flexible residues with other amino acids has been used to improve the thermostability of many enzymes [36]. Fang et al. [21] screened two mutants of ZENG with higher thermostability, S162P and S220R through MD simulation, then constructed the double sites mutant, S162P/S220R, which had more than 90% residual activity at 55 °C for 10 min.

**Figure 6:**
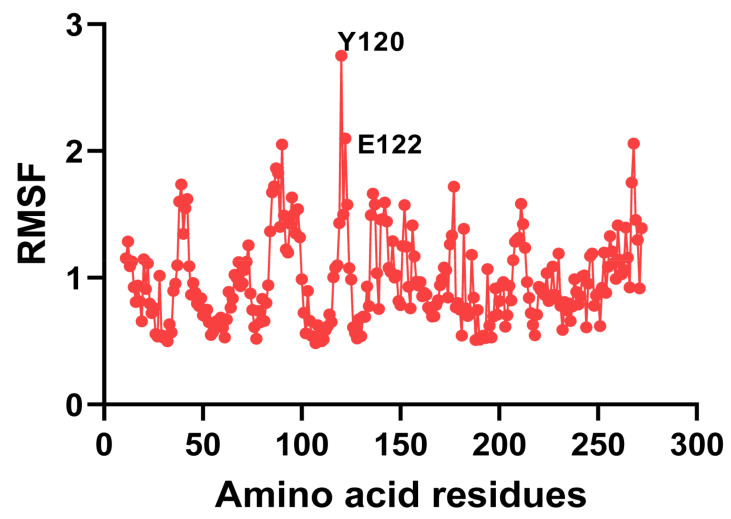
The RMSF results of amino acid residues in ZHRnZ.

Here the Discovery Studio 2019 software was used for MD simulation, and the “Analyze the Trajectory” module was used to calculate the RMSF value of each amino acid residue in ZHRnZ (Figure 6). The first two hotspots, Tyr120 and Glu122, were selected as tentative mutation sites. The mutation energy and stability of each mutant after saturated mutations at the two hotspots were calculated by the “Calculate the Mutation Energy (Stability)” function of Discovery Studio 2019 (Table 2). According to the prediction, mutations at Tyr120 were abandoned because all mutants at the site were destabilizing or neutral. Finally, 14 stabilizing mutation directions at Glu122 were determined for the following experiments. As shown in Figure 2B, the mutation site Glu122 was far from the catalytic center. Therefore, it was speculated that the mutations may not affect the enzyme-substrate interaction.

**Figure 7:**
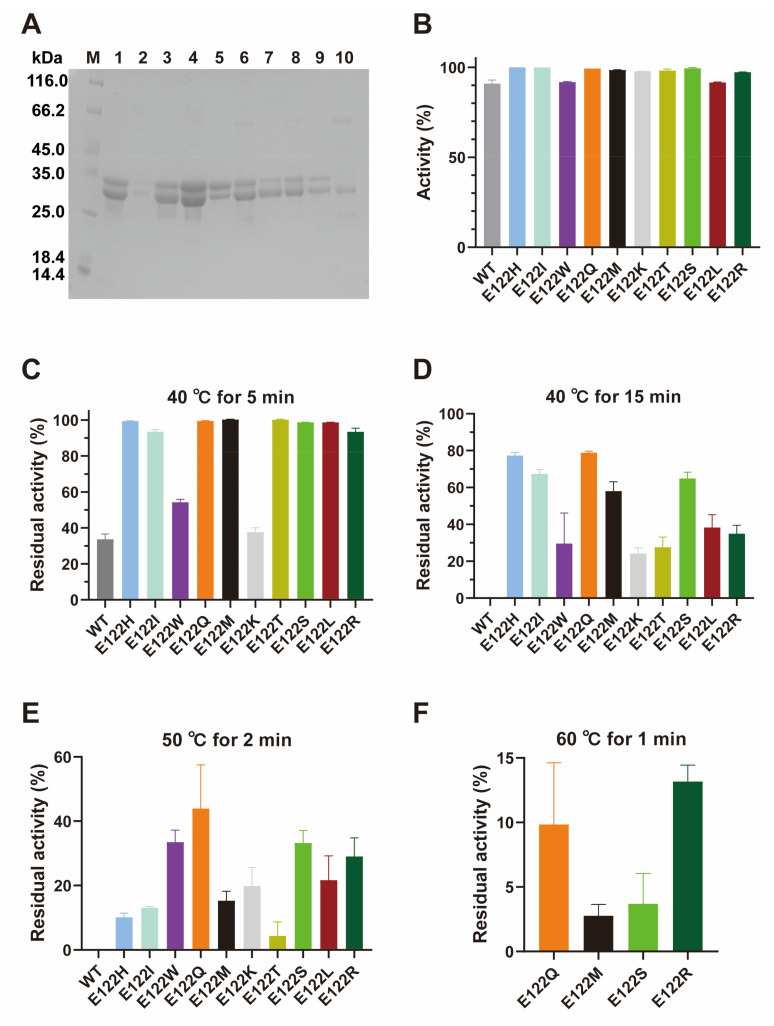
The characteristics of the mutants. (**A**) SDS-PAGE analysis of mutants. Lane M represents the protein ladder (116.0, 66.2, 45.0, 35.0, 25.0, 18.4, and 14.4 kDa); Lanes 1–10 were mutants E122I, E122H, E122M, E122Q, E122R, E122L, E122K, E122S, E122W, and E122T, respectively. (**B**) The effect of WT and mutants on the degradation of ZEN. (**C**) The residual activity of WT and mutants at 40 °C for 5 min. (**D**) The residual activity of WT and mutants at 40 °C for 15 min. (**E**) The residual activity of WT and mutants at 50 °C for 2 min. (**F**) The residual activity of mutants E122Q, E122M, E122S, and E122R at 60 °C for 1 min.

Mutants E122H, E122I, E122W, E122Q, E122M, E122K, E122T, E122S, E122L, and E122R were successfully constructed and expressed in *E. coli* in this study as shown in Figure 7A. The activity of the 10 mutants in degrading ZEN was all above 90% (10 μg·mL^−1^ ZEN, 25 μg·mL^−1^ enzyme, pH 9.0, 45 °C, 15 min), which means that these were positive mutations of ZHRnZ (Figure 7B). The residual activity of the mutants except for E122W and E122K was more than 90% at 40 °C for 5 min, which was approximately three times higher than that of the wild-type (WT, 34%) as shown in Figure 7C. The activity of mutants E122H, E122I, E122Q, and E122S was relatively stable, with more than 60% of their retained activity at 40 °C for 15 min, while the WT lost its activity (Figure 7D). As shown in Figure 7E, mutant E122Q still displayed an obvious advantage (44% residual activity) at 50 °C for 2 min. Moreover, mutants E122W, E122S, and E122R also retained more than 30% of their initial activity at 50 °C for 2 min. Considering the advantages, mutants E122Q, E122M, E122S, and E122R were selected to test the residual activity at 60 °C for 1 min. It was seen that only mutants E122Q and E122R kept approximately 10% of their initial activity (Figure 7F). These results suggested that the thermostability of all mutants, especially that of E122Q and E122R, had been greatly improved.

### 2.5. The Enzymatic Kinetic Parameters of WT, E122Q and E122R

The *K*_m_, *V*_max_, *K*_cat_, and *K*_cat_/*K*_m_ were calculated with GraphPad Prism 8.0.1 software. The enzymatic kinetic parameters of WT and the two mutants, E122Q and E122R, are shown in Table 3. The *K*_m_, *V*_max_, and *K*_cat_ of WT were 21.51 μg·mL^−1^, 0.06051 μg·mL^−1^·s^−1^, and 0.00242 s^−1^, respectively. The *V*_max_ of mutant E122Q was three times higher than that of the WT, but the affinity of E122Q to ZEN was a quarter of the WT. The *K*_m_ value of the WT to ZEN increased from 21.51 to 86.38 after 122Glu mutated to 122Gln, indicating that the affinity of E122Q to ZEN was decreased. Overall, the catalytic efficiency (*K*_cat_/*K*_m_) of E122Q to ZEN was slightly worse than that of the WT. However, the *K*_m_ value of E122R was 8.123, which means the affinity of E122R to ZEN was 2.5 times higher than that of the WT, and the *V*_max_ was half of the WT. The catalytic efficiency of E122R was 1.3 times higher than that of the WT. Commonly, the mutation causes a change in enzymatic kinetic parameters. Wang et al. [28] fused a segment of amphiphilic short peptide S1 at the N-terminus of Zhd11D, which surprisingly resulted in both improved activity (1.5-fold) and thermostability (2-fold at 40 °C). The catalytic efficiency of S1-Zhd11D was 1.44 times higher than that of Zhd11D. The catalytic efficiency of S162P/S220R was 1.66 times higher than that of the wild-type of ZENG [21].

## 3. Materials and Methods

### 3.1. Plasmids, Strains and Chemicals

The plasmids pET-28a and the receptive *E. coli* BL21 (DE3) were purchased from Tsingke (Beijing, China). ZEN, α-ZOL, β-ZOL, and α-ZAL were purchased from Pribolab (Qingdao, China). The synthesis of primers and the Sanger sequencing of DNA were performed by Sangong (Shanghai, China). The isopropyl-β-D-thiogalactopyranoside (IPTG) was purchased from Biotopped (Beijing, China). The other regents are high-performance liquid chromatography (HPLC) grade and analytical grade, respectively.

### 3.2. Bioinformatics Analysis and Molecular Docking

The ZHD101 protein sequence (Accession No. BAC02717.1) was used as the template for comparison in NCBI protein BLAST. A sequence with high similarity was selected and then multiple sequence alignment was performed by ClustalW (https://www.genome.jp/tools-bin/clustalw (accessed on 2 September 2024)) with ZHD101 as a template. The visualization was performed using Espript 3.0 (https://espript.ibcp.fr/ESPript/cgi-bin/ESPript.cgi (accessed on 2 September 2024)). The structure of ZHD101 was retrieved from the RSCB Protein Data Bank (PDB ID: 3WZL) and was used as the template. The tertiary structure of the candidate proteins was obtained through SWISS-MODEL (https://swissmodel.expasy.org/ (accessed on 2 September 2024)) homologous modeling. The presence of signal peptides of ZHRnZ was predicted with Signal 5.0 (https://services.healthtech.dtu.dk/services/SignalP-5.0/ (accessed on 2 September 2024)). The molecular weight and isoelectric point of ZHRnZ were calculated by Expasy (https://web.expasy.org/protparam/ (accessed on 2 September 2024)). The molecular docking between ZHRnZ and ZEN was performed with AutoDock Vina 1.2.3, and the results were visualized using Discovery Studio 2019.

### 3.3. Gene Cloning, Expression and Purification of Recombinant ZHRnZ

To obtain the expression vector pET28a-ZHRnZ, the optimized ZHRnZ gene segment was cloned into the pET28a vector between the BamHI and XhoI enzyme restriction sites. The resulting recombinant plasmid was transformed into *E. coli* BL21 (DE3) competent cells via the heat shock method. The target protein with a His-tag can be expressed.

The *E. coli* BL21 cells harboring the genes of ZHRnZ were grown in Luria-Bertani (LB) solid medium containing 25 μg·mL^−1^ kanamycin for 12 h at 37 °C. Subsequently, a single colony was inoculated into a 5 mL LB liquid medium containing 25 μg·mL^−1^ kanamycin and cultured overnight (37 °C, 180 rpm). Then, the 5 mL fermentation liquid was inoculated into a 500 mL fresh LB medium for further culturing. IPTG (0.4 mM) was added to the medium when the optical density at 600 nm reached 0.6–0.8. The culture was induced for 12–16 h (16 °C, 120 rpm). After centrifugation at 3500× *g* for 25 min at 4 °C, the bacterial cell was collected. The cell was suspended in 25 mM Tris-HCl buffer (pH 7.0) and re-centrifuged (4 °C, 3500× *g*, 15 min). After re-suspending 3 times, the cell was suspended in a binding buffer (25 mM Tris, 150 mM NaCl, and 5 mM Imidazole, pH 7.5). The cell disruption was performed with 1 mM phenyl methane sulfonyl fluoride (PMSF) by sonification in an ice water bath (power 300 W, ultrasound for 3 s, pause for 5 s, working for 10 min, repeated 3 times). The cell debris was removed by centrifugation (12,000× *g*, 10 min, 4 °C), then the supernatant was filtered by a 0.45 μm filter membrane. The recombinant protein was purified by a Ni^2+^-NTA affinity chromatography column (Sangong, Shanghai, China). The collected fractions were concentrated in ultrafiltration tubes with 10 kDa of molecular weight cutoff (Sigma-Aldrich, St. Louis, MO, USA), and the remaining imidazole was removed. The concentrated protein was analyzed with SDS-PAGE and a BCA Protein Quantitative Analysis Kit (Aidlab, Beijing, China) was used to determine the concentration.

### 3.4. Enzymatic Activity Assay

The enzyme activity of recombinant ZHRnZ was measured by the degradation of ZEN and its derivatives. The ZEN degradation rate of the recombinant ZHRnZ was determined in 200 μL of Tris-HCl buffer (50 mM, pH 7.5) containing 25 μg·mL^−1^ of recombinant ZHRnZ and various final concentrations of ZEN (2, 10, 20 μg·mL^−1^) after incubation at 37 °C for different times (2, 5, 10, 15, 30, 60 min). The mixtures were added to equal volumes of methanol to end the reactions. A total of 200 μL of Tris-HCl buffer (50 mM, pH 9.0) containing 25 μg·mL^−1^ of recombinant ZHRnZ and 10 μg·mL^−1^ of toxins (ZEN, α-ZOL, β-ZOL, and α-ZAL) were incubated at 45 °C for 15 min. The reactions were terminated by adding an equal amount of methanol. The control was prepared in the absence of recombinant ZHRnZ.

### 3.5. Enzymatic Characteristics of Recombinant ZHRnZ

The effect of pH on recombinant ZHRnZ activity towards ZEN was determined by incubating in different pH buffers (pH 3.0–11.0) at 37 °C. The buffers used in the tests contained 50 mM disodium hydrogen phosphate-citrate buffer (pH 3.0–6.0), 50 mM Tris-HCl buffer (pH 7.0–10.0), and 50 mM sodium hydroxide-glycine buffer (pH 11.0). The effect of the temperature of recombinant ZHRnZ activity towards ZEN was determined by incubating at different temperatures (30, 37, 40, 42, 45, 47, 50, 60, 70 °C) in pH 9.0 buffer.

The acid resistance of recombinant ZHRnZ was studied by incubating the purified enzyme at 4 °C in different pH buffers (pH 4.0–9.0) for a range of times (1, 6, 12, 24, 48 h), followed by measuring the residual activities for ZEN degradation under the optimal condition (pH 9.0, 45 °C). To test the thermostability of recombinant ZHRnZ, the purified enzyme was incubated at different temperatures (30, 40, 50 °C) for different times (2, 5, 10, 15, 20 min). The remaining activities were measured with ZEN at 45 °C and pH 9.0.

All reactions were performed in a 200 μL buffer containing 25 μg·mL^−1^ of ZHRnZ and 10 μg·mL^−1^ of ZEN. The control was prepared in the absence of recombinant ZHRnZ. After incubation for 15 min, an equal volume of methanol was added to terminate the reaction.

### 3.6. Single Point Mutation of ZHRnZ

MD simulation was performed using Discovery Studio 2019. The 50 ns movement of the proteins was simulated with CHARMm36 force field and TIP3P water molecule type at 300 K, and the results were measured every 2 ps. The RMSF value was calculated using the Analyze the Trajectory module. Two residues with the largest RMSF value were selected as mutation sites. The Mutation Energy (Stability) function of Discovery Studio 2019 was used to calculate the mutation energy of each mutant after saturated mutation at the selected site, and the mutants with stabilizing mutation directions were selected for subsequent experiments.

The mutant primers were designed with Snapgene and the mutant plasmids were constructed with Tiangen KM101 kit (Beijing, China). The mutant primers are shown in Table 4. The *E. coli* BL21 (DE3) competent cells were transformed with the mutant plasmids via the heat shock method. Each mutant was expressed and purified. To determine the ZEN degradation of mutants, 25 μg·mL^−1^ of enzyme and 10 μg·mL^−1^ of ZEN was incubated in a 200 μL reaction system for 15 min under optimal conditions (pH 9.0, 45 °C). To test the thermostability of mutants, the enzymes were treated at 40 °C for 5, 15 min, and 50 °C for 2 min, respectively. The residual activities were measured with ZEN at 45 °C and pH 9.0. Several promising mutants were selected to determine the residual activities after treatment at 60 °C for 1 min. The control groups were prepared in the absence of enzymes. All the reactions were terminated by adding equal volumes of methanol after incubation for 15 min.

### 3.7. The Kinetic Parameters of Enzymatic Reactions of Recombinant ZHRnZ, E122Q and E122R

To obtain the kinetic parameters of enzymatic reactions of recombinant ZHRnZ, E122Q, and E122R, different concentrations of ZEN (10, 20, 40, 50, 60 μg·mL^−1^) and 25 μg·mL^−1^ of recombinant ZHRnZ were added into a 200 μL reaction system, and incubated for 10 min (pH 9.0, 45 °C). An equal volume of methanol was added to terminate the reaction.

### 3.8. Determination of ZEN

All reactions were carried out in three replicates and the concentration of residual ZEN was determined via the HPLC system (Shimadzu LC-10 AT, Shimadzu, Tokyo, Japan) equipped with fluorescence detection (SPD-20A, Shimadzu, Tokyo, Japan) and a Diamonsil^®^ C18 reverse phase column (150 mm × 4.6 mm, 5 μm, Dikma, Beijing, China). Terminated reaction mixtures of ZEN were injected into the HPLC system after being filtered by a 0.22 μm filter. The injection volume was programmed to 20 μL and ZEN was successfully detected by fluorescence detection (λ_ex_ = 274 nm, λ_em_ = 440 nm). The mobile phase consisted of acetonitrile and water (6:4, *v*/*v*), with a flow rate of 1.0 mL·min^−1^.

The degradation rate, relative activity, and residual activity were calculated with the following equation: Degradation rate (%) = (Control − Sample)/Control × 100%; Relative activity (%) = Degradation rate of each group/Degradation rate of the group with the highest degradation rate × 100%; Residual activity (%) = Degradation rate of treated group/Degradation rate of untreated group × 100%.

### 3.9. Statistical Analysis

The results were calculated with GraphPad Prism 8.0.1 and expressed as the mean ± standard error of the mean (SEM) of the three independent determinations. Each experiment was repeated at least twice to verify the results.

## 4. Conclusions

In summary, a new zearalenone lactone hydrolase ZHRnZ from *Rosellinia necatrix* was characterized in this study, which demonstrated good degradation ability on ZEN and its derivatives α-ZOL, β-ZOL, and α-ZAL. The optimum pH for ZHRnZ was observed at pH 9.0 with 90% of ZEN degradation, and more than 60% of ZEN was degraded at pH 6.0–10.0. The recombinant ZHRnZ also had an over 60% relative activity towards ZEN at 30–50 °C, while the optimal temperature was 45 °C. We obtained 10 mutants of ZHRnZ with improved thermostability by single-point mutation. Specifically, mutants E122Q and E122R significantly improved the thermostability, which retained 44% and 30% of their initial activity after treatment at 50 °C for 2 min, respectively, and kept approximately 10% of their initial activity at 60 °C for 1 min. The enzymatic kinetic analysis showed that the catalytic efficiency of E122R was 1.3 times higher than that of the WT. After comprehensive consideration, mutant E122R was selected as a prospective enzyme preparation to detoxify ZEN in food and feed in the future.

## Figures and Tables

**Table 1:** Characteristics of partial ZEN lactone hydrolase.

Enzyme	Source	Optimal Temperature (°C)/pH	Thermostability	References
ZHD_LD	*Exophiala spinifera*	50/7.0–10.0	NR ^a^	[16]
ZenH	*Aeromicrobium* sp. HA	55/7.0	Lost activity at 40 °C for 2 min	[17]
ZENG	*Gliocladium roseum*	38/7.0	Retained 20% residual activity at 48 °C for 7 min, 10% at 53 °C for 2 min	[18]
H134L/S136L	*Gliocladium roseum*	NR	Retained 50% residual activity at 48 °C for 7 min, 50% at 53 °C for 2 min	[18]
H134F/S136F	*Gliocladium roseum*	NR	Retained 70% residual activity at 48 °C for 7 min, 44% at 53 °C for 2 min	[18]
S162P/S220R	*Gliocladium roseum*	NR	Retained 90% residual activity at 55 °C for 10 min	[21]
ZHD607	*Phialophora americana*	35/8.0	Retained 56% residual activity at 40 °C for 10 min, 20% for 30 min	[22]
TRI	*Trichoderma aggressivum*	40/9.5	Retained 25% residual activity at 50 °C for 10 min, 20% at 65 °C for 2 min	[23]
ZENC	*Neurospora crassa*	45/8.0	Lost activity at 60 °C for 1 min	[24]
ZHD-P	*Trichoderma agressivum*	45/7.5–9.0	Remained activity at 25–40 °C for 1 h, retained 40% residual activity at 45 °C for 1 h	[25]
ZENY	*Bacillus subtilis* YT-4	37/8.0	Retained 50% residual activity at 45 °C for 10 min, 40% at 50 °C for 10 min	[26]
NΔ11	*Bacillus subtilis* YT-4	NR	Retained 63% residual activity at 45 °C for 10 min, 45% at 50 °C for 10 min	[26]
N5V	*Bacillus subtilis* YT-4	NR	Retained 77% residual activity at 45 °C for 10 min, 50% at 50 °C for 10 min	[26]
CbZHD	*Cladophialophora batiana*	35/8.0	Retained 40% residual activity at 40 °C for 2 min	[27]
Zhd11D	*Phialophora attinorum*	35/8.0	Retained 40% residual activity at 40 °C for 20 min, 15% for 60 min	[28]
S1-Zhd11D	*Phialophora attinorum*	35/8.0	Retained 75% residual activity at 40 °C for 20 min, 35% for 60 min	[28]
Zhd518	*Rhinocladiella mackenziei*	40/8.0	Remained activity at 20–40 °C for 30 min, retained 25% residual activity at 45 °C for 10 min	[29]
ZHRnZ	*Rosellinia necatrix*	45/9.0	Retained 42% residual activity at 40 °C for 5 min, lost activity at 50 °C for 2 min	this study
E122Q	*Rosellinia necatrix*	NR	Retained 79% residual activity at 40 °C for 15 min, 44% at 50 °C for 2 min	this study
E122R	*Rosellinia necatrix*	NR	Retained 29% residual activity at 50 °C for 2 min, 13% at 60 °C for 1 min	this study

^a^ NR = not reported.

**Table 2:** Prediction of mutants at Glu122 and Tyr120 sites.

Mutation	Mutation Energy	Effect of Mutation
A:GLU122 > ARG	−0.77	STABILIZING
A:GLU122 > CYS	−1.47	STABILIZING
A:GLU122 > GLN	−1.3	STABILIZING
A:GLU122 > HIS	−1.05	STABILIZING
A:GLU122 > ILE	−1.69	STABILIZING
A:GLU122 > LEU	−0.97	STABILIZING
A:GLU122 > LYS	−0.68	STABILIZING
A:GLU122 > MET	−0.96	STABILIZING
A:GLU122 > PHE	−1.1	STABILIZING
A:GLU122 > SER	−0.99	STABILIZING
A:GLU122 > THR	−1.28	STABILIZING
A:GLU122 > TRP	−1.72	STABILIZING
A:GLU122 > TYR	−0.91	STABILIZING
A:GLU122 > VAL	−1.3	STABILIZING
A:GLU122 > ALA	−0.39	NEUTRAL
A:GLU122 > ASN	−0.46	NEUTRAL
A:GLU122 > ASP	0.21	NEUTRAL
A:GLU122 > GLU	0.02	NEUTRAL
A:GLU122 > GLY	0.25	NEUTRAL
A:TYR120 > CYS	0.45	NEUTRAL
A:TYR120 > LEU	0.02	NEUTRAL
A:TYR120 > PHE	0.04	NEUTRAL
A:TYR120 > TRP	−0.2	NEUTRAL
A:TYR120 > TYR	0.01	NEUTRAL
A:GLU122 > PRO	3.46	DESTABILIZING
A:TYR120 > ALA	1.56	DESTABILIZING
A:TYR120 > ARG	1.88	DESTABILIZING
A:TYR120 > ASN	1.39	DESTABILIZING
A:TYR120 > ASP	2.14	DESTABILIZING
A:TYR120 > GLN	1.23	DESTABILIZING
A:TYR120 > GLU	2.78	DESTABILIZING
A:TYR120 > GLY	2.6	DESTABILIZING
A:TYR120 > HIS	0.83	DESTABILIZING
A:TYR120 > ILE	0.6	DESTABILIZING
A:TYR120 > LYS	1.51	DESTABILIZING
A:TYR120 > MET	1.21	DESTABILIZING
A:TYR120 > PRO	8.02	DESTABILIZING
A:TYR120 > SER	1.69	DESTABILIZING
A:TYR120 > THR	1.47	DESTABILIZING
A:TYR120 > VAL	0.72	DESTABILIZING

**Table 3:** The kinetic parameters of enzymatic reactions of recombinant ZHRnZ and mutants E122Q, E122R.

Enzyme	*K*_m_ (μg·mL^−1^)	*V*_max_ (μg·mL^−1^·s^−1^)	*K*_cat_ (s^−1^)	*K*_cat_/*K*_m_ (s^−1^·μg^−1^·mL)
WT	21.51	0.06051	0.00242	1.13 × 10^−4^
E122Q	86.38	0.1853	0.00741	0.86 × 10^−4^
E122R	8.123	0.03004	0.001202	1.48 × 10^−4^

**Table 4:** The primers of mutants.

Mutant	Forward Primer (5′-3′)	Reverse Primer (5′-3′)
E122H	CGCAGCGCACGCGATGCGGATAACCCGCC	GGCGGGTTATCCGCATCGCGTGCGCTGCG
E122I	GCAGCGCACGCGTATCGGATAACCCGCC	GGCGGGTTATCCGATACGCGTGCGCTGC
E122W	GGCGGGTTATCCGTGGCGCGTGCGCTGCG	CGCAGCGCACGCGCCACGGATAACCCGCC
E122Q	GGCGGGTTATCCGCAGCGCGTGCGCTGCGC	GCGCAGCGCACGCGCTGCGGATAACCCGCC
E122M	GGCGGGTTATCCGATGCGCGTGCGCTGCGC	GCGCAGCGCACGCGCATCGGATAACCCGCC
E122K	GTGGCGGGTTATCCGAAACGCGTGCGCTGC	GCAGCGCACGCGTTTCGGATAACCCGCCAC
E122T	GGCGGGTTATCCGACCCGCGTGCGCTGCGC	GCGCAGCGCACGCGGGTCGGATAACCCGCC
E122S	GTGGCGGGTTATCCGAGCCGCGTGCGCTGCGC	GCGCAGCGCACGCGGCTCGGATAACCCGCCAC
E122L	GTGGCGGGTTATCCGCTGCGCGTGCGCTGCGC	GCGCAGCGCACGCGCAGCGGATAACCCGCCAC
E122R	GTGGCGGGTTATCCGCGTCGCGTGCGCTGCGC	GCGCAGCGCACGCGACGCGGATAACCCGCCAC

## Data Availability

The original contributions presented in the study are included in the article, further inquiries can be directed to the corresponding author.
